# The problem of organ transplantation based on three-sided stable matching

**DOI:** 10.1371/journal.pone.0341764

**Published:** 2026-07-08

**Authors:** Ruya Fan, Yan Chen, Bo Shan

**Affiliations:** 1 School of Management, Shenyang University of Technology, Shenyang, China; 2 School of Science, Shenyang University of Technology, Shenyang, China; Istinye University: Istinye Universitesi, TÜRKIYE

## Abstract

Aiming at the problem of matching scarce resources among donors, recipients, and medical institutions in organ transplantation, a stable three-sided matching method is proposed. Firstly, in view of the preference structure characteristics of the problems in the context of organ transplantation, a mixed preference structure from the three-sided matching problem is introduced for description, and the stability conditions under this structure are also provided, intuitionistic fuzzy information was utilized to quantify uncertain indicators during organ transplantation. The Erlang distribution was introduced to describe the randomness of the occurrence of organ donors. Coping strategies for possible false reporting behaviors of organ recipients were designed, and a three-sided matching model of donors, recipients, and medical institutions was constructed. And proved the stability of this matching; Secondly, based on the NSGA-III algorithm, the initial search strategy of the Bird Swarm algorithm was introduced, a new mutation method was designed, and its feasibility was theoretically analyzed. Finally, the feasibility of the model and algorithm was verified through simulation examples in the context of organ transplantation, and the performance of the algorithm was analyzed and verified in combination with algorithm complexity, effectiveness, and ablation experiments. The experimental results show that the model and algorithm can effectively improve the stability of the matching results and increase the efficiency of resource allocation.

## 1. Introduction

Organ transplantation refers to the process of excising part or the entirety of an organ from a donor and implanting it into the recipient’s body to replace a damaged or failing organ. This procedure involves multiple dimensions, including the allocation of medical resources, the extension of patients’ lives, considerations of medical ethics, and societal development needs. With continuous advancements in medical technology and ongoing in-depth research, the scope of organ transplantation is expected to further expand, positioning it as one of the critical therapeutic approaches for treating various end-stage diseases [1], thereby offering renewed hope and extended life to more transplant recipients. According to the latest report released by the China Organ Transplant Development Foundation—the *China Organ Transplant Developme*nt Report (2024)—China ranks second in the world in terms of organ transplant volume, with postoperative survival rates continually improving. Organ transplantation not only saves the lives of recipients and enhances their quality of life but also contributes to social harmony and development. However, the field still faces numerous challenges and issues.

Apart from the complex ethical and legal issues involved in organ donation and transplantation [1], the core issue of organ transplantation lies more in the matching and allocation of scarce resources among multiple participants [2]. Traditionally, most studies focus on two‑sided matching between donors and recipients, aiming to improve the success rate and quantity of transplantation [[href:#_ENREF_3]^3^- ^5^]. Clinical health prediction-related research has generally recognized the importance of integrating data from multiple medical institutions and has regarded medical institutions as key analysis scenarios [6]. Inspired by this, the study no longer confines itself to the traditional donor-receiver two-sided matching framework but formally incorporates medical institutions as the core decision-making entity in the matching system and constructs a stable matching model involving three parties: organ donors, organ recipients, and medical institutions.

**Fig 1:**
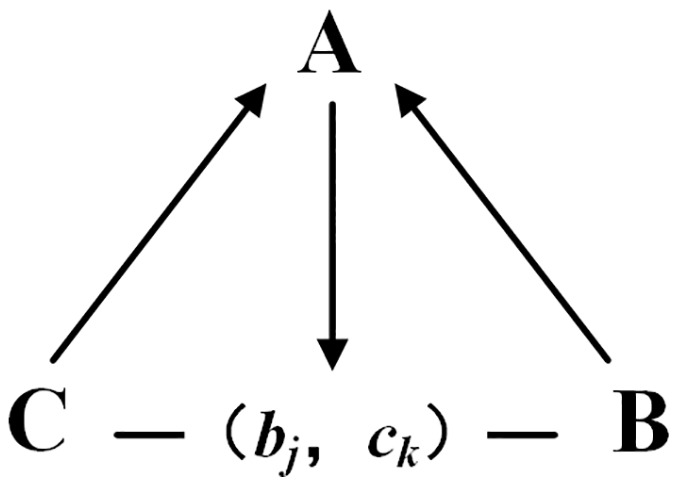
Tripartite Relationship with Hybrid Preferences.

The preferences and decision‑making objectives of the three parties are heterogeneous: The organ recipients need to consider both the cost (including medical costs and time costs) and the medical level when choosing a medical institution. Since there are currently cases of live organ donation in organ transplantation, the organ donors also need to consider the medical level of the medical institution during the surgery for the organ donors. The medical institution mainly judges whether the organ recipient is suitable for the surgery based on two indicators: whether the matching between the organ recipient and the organ donor is successful, and the severity of the condition and the recovery after the surgery. Therefore, the issue of organ transplant matching is essentially a typical three-sided matching problem with a hybrid preference structure(Let $A=\left\{a_{\text{1}},a_{\text{2}},\cdots a_{i}\right\}$, $B=\left\{b_{\text{1}},b_{\text{2}},\cdots b_{j}\right\}$, $C=\left\{c_{\text{1}},c_{\text{2}},\cdots c_{k}\right\}$(where *i*, *j*, *k* are positive integers, respectively) represent the sets and individuals among the three parties, Ω=A×B×C. The element *a*_*i*_ in subject *A* has a certain preference for the pair $\left(b_{j},c_{k}\right)$ in subject B×C; the element *b*_*j*_ in subject *B* has a certain preference for the element *a*_*i*_ in subject *A*; and the element *c*_*k*_ in subject *C* has a certain preference for the element *a*_*i*_ in subject *A*. This constitutes a hybrid preference structure, as shown in Fig 1 below).

This study introduces a hybrid preference three-sided stable matching framework to solve the matching optimization problem involving three parties: organ donors, organ recipients, and medical institutions. Intuitionistic fuzzy information is used to quantify uncertain indicators, the Erlang distribution is used to describe the randomness of donor occurrence, and a penalty mechanism is designed to suppress the false reporting behavior of recipients. On this basis, a three‑sided stable matching model of donors, recipients, and medical institutions is constructed. Furthermore, an improved NSGA‑III algorithm is proposed by integrating the initial search strategy of the bird swarm algorithm and a new adaptive mutation operator. Finally, simulation experiments verify the effectiveness and stability of the model and algorithm.

The remaining sections of this study are structured as follows: Section 2 conducts a literature review of the existing research; Section 3 addresses issues such as data uncertainty, the randomness of events, and possible false statements by organ recipients, proposes corresponding solutions, and proves the stability conditions for the stable matching of the three-sided; Section 4 presents a three-sided matching framework involving organ recipients, medical institutions, and organ donors, and constructs a three-sided stable matching model applicable to organ transplantation. Section 5 designs an improved NSGA-III algorithm to solve the proposed problems. Section 6 uses specific numerical examples to evaluate the performance and computational complexity of the model and algorithm solutions. Section 7 summarizes the results and shortcomings of this study and outlines the directions for future research.

## 2. Literature review

Organ transplantation is a typical resource allocation problem involving multiple stakeholders. The focus of the research is not only on increasing the number of transplants, but also on ensuring the stability, efficiency, and multi-party coordination of the transplants. This paper reviews the existing research from four aspects: two-sided organ matching models, random and uncertain matching, multi-objective optimization algorithms, and three-sided stable matching theory, and summarizes the key research gaps.

Early studies focused on the donor–recipient two-sided matching and aimed to maximize the number of transplants and success rates. Dickerson et al.[3] proposed a failure-aware mechanism to reduce post-allocation matching failures. Anderson et al.[2] constructed an integer programming model to restrict each donor and recipient to at most one transplant. Blum et al.[4] and Smeulders et al.[5] used graph theory and two-stage stochastic programming to optimize kidney exchange efficiency.

With the development of organ donation and transplantation, scholars began to consider multi-objective optimization and heterogeneous preferences. Carvalho et al.[7] proposed a robust model to reduce the cost of matching adjustment. Arikan et al.[8] found that organ quality, waiting time, and competition affect recipients’ acceptance willingness. Arora and Subramanian [9] modeled the organ donation chain involving hospitals and procurement organizations. Hasankhani and Khademi [10] improved heart allocation rules by introducing dynamic priorities. However, almost all these studies treat the system as a donor–recipient two-sided problem and completely ignore the core role of medical institutions in registration, evaluation, surgery, and allocation. The matching decision-making mechanism is incomplete.

Randomness and uncertainty are inherent in organ transplantation and significantly affect matching stability and efficiency. Su and Zenios [11] addressed information asymmetry and incentive issues in organ allocation. Van De Klundert et al.[12], You and Vossen [13] used queuing models and Markov processes to describe waiting time and dynamic arrival. Blum et al.[4], Bandi et al.[14], and Aouad and Saritaç [15] modeled the random arrival and dropout of donor–recipient pairs. Perlman et al.[16] balanced fairness and transplant utility through a Markov process. However, the existing research has obvious limitations: The ambiguity of the preference expressions of the matching subjects, the randomness of the subjects’ arrival, and the deceptive preference behaviors of the matching subjects have not been systematically addressed within a unified framework.

Algorithmic innovation is key to solving large-scale matching problems. Santos et al.[17] developed a kidney exchange simulator. Le et al.[18] optimized dual-donor exchange chains. Papalexopoulos et al.[19] combined machine learning and integer programming for dynamic allocation policy design. Delorme et al.[20], Alvelos et al.[21], and Lam and Mak-Hau [22] used diving and branch-and-price algorithms to improve solution efficiency. Blom et al.[23] and Klimentova et al.[[href:#_ENREF_24]^24^, ^25^] considered matching stability and multi-agent integer programming models. These algorithms improve solving efficiency but are mostly designed for two-sided or kidney-exchange structures. They cannot be directly applied to three-sided stable matching with hybrid preferences.

Three-sided stable matching was first proposed by Knuth [26]. Alkan [27] showed that stable matching may not exist in general three-sided systems. Danilov [28] provided sufficient conditions for the existence of stable matching in multi-sided systems. Ng and Hirschberg [29] proved that verifying three-sided stability is NP-hard. Zhang et al.[30] confirmed that stable matching exists under a hybrid preference structure, which lays a theoretical foundation for this paper. However, the three-sided matching theory is rarely applied in the field of organ transplantation. Currently, there is a lack of three-sided stable matching models and algorithms suitable for transplantation scenarios.

At present, there are still three key deficiencies in the relevant research: (1) Most studies only consider the bilateral relationship between donors and recipients, ignoring the key role of medical institutions as the matching hub; (2) The uncertainty of indicator quantification, the randomness of donor occurrence, and the potential false reporting of recipients are rarely considered comprehensively; (3) There is a lack of targeted research on three‑sided stable matching for organ transplantation scenarios.

To fill these gaps, this study makes some contributions as follows: (1) A hybrid preference three‑sided matching model is constructed for organ transplantation, covering recipients, medical institutions, and donors; (2) Uncertainty, randomness, and information false reporting are systematically processed to improve the rationality and stability of matching; (3) An improved NSGA‑III algorithm is designed to enhance population diversity, convergence, and solution stability.

## 3. Problem description

### 3.1. Intuitionistic fuzzy sets

According to the organ matching process mentioned in the first part of this study, factors such as medical level, severity of the disease, and post-operative recovery play a crucial role in determining whether the transplantation process can proceed smoothly. However, for organ recipients and medical institutions, it is difficult to provide a completely precise quantitative assessment of these indicators. Therefore, the intuitionistic fuzzy set theory was introduced to handle this fuzziness and uncertainty, thereby improving the accuracy and sensitivity of the matching results.

**Definition 1** [31]Let *X* be a non-empty set. For an element *x* belonging to *X*, the membership degree is denoted as $\mu_{A}\left(x\right)\subset \left[0,1\right]$, and the non-membership degree is denoted as $v_{A}\left(x\right)\subset \left[0,1\right]$. It is stated that


$$\begin{array}{c} A=\left\{\left\langle x,\mu_{A}\left(x\right),v_{A}\left(x\right)\right\rangle |x\in X\right\} \end{array}$$ (1)

is an interval-valued intuitionistic fuzzy set. Moreover, $1-\mu_{A}\left(x\right)-v_{A}\left(x\right)$ represents the hesitancy degree of element *x* in *X* belonging to the set *A*.

By comprehensively considering the relationships among membership degree, non-membership degree, and hesitancy degree, the application of a score function to the intuitionistic fuzzy decision matrix enables more precise and reasonable calculation results.

**Definition 2** [32] Let α=([a,b],[c,d]) be an interval-valued intuitionistic fuzzy number. Then the functionΔ(α)=(a−c+b−d)/2is called the score function of α, where [a,b]⊂[0,1], [c,d]⊂[0,1], b+d≤1.

Intuitionistic fuzzy decision matrices serve as a significant tool in the field of decision analysis. By integrating three dimensions—membership degree, non-membership degree, and hesitancy degree—they comprehensively represent uncertainty, thereby aligning decisions more closely with real-world scenarios [[href:#_ENREF_33]^33^, ^34^]. Constructing an intuitionistic fuzzy decision matrix for the tripartite stable matching problem in organ transplantation can be divided into the following steps:

**Step 1:** Collect evaluation information from decision-makers on the three indicators—medical level, severity of illness, and postoperative recovery—and construct an intuitionistic fuzzy decision matrix that incorporates hesitancy degrees.

**Step 2:** Calculate the score functions based on the interval-valued intuitionistic fuzzy numbers in the intuitionistic fuzzy decision matrix.

**Step 3:** Rank the score functions according to their numerical values to represent the decision-makers’ preferences.

### 3.2. Randomness

In this section, randomness mainly refers to the irregular arrival times of organ donors and the random waiting times for organ recipients to obtain suitable donors, which cannot be accurately predicted in advance. The fundamental reason for the randomness in the organ transplantation process is that the appearance of donors is uncertain. Organ donation is affected by actual conditions such as regional medical resources, donation rates, transportation, and weather, showing obvious spatial and temporal randomness, which is not fixed or predictable. At the same time, the waiting times for each stage of organ transplantation are random. The waiting time for recipients to obtain matching organs is random and non-Markovian, which directly affects the timeliness of treatment and the stability of matching.

The *k*‑stage Erlang distribution is adopted to describe the randomness in the organ transplantation process. This is mainly because the waiting time for the recipient to obtain a suitable donor shows distinct multi-stage, non-sequential, and random characteristics, which cannot be accurately described by the traditional exponential distribution. As the sum of *k* independent and identical exponential variables, the Erlang distribution can well fit the entire process of organ transplantation consisting of multiple consecutive medical stages, and is naturally adapted to the queuing system model constructed in this study. At the same time, it helps to discretize the continuous-time stochastic process into static decision moments, laying a mathematical foundation for proving the existence of three-sided stable matching. Thus, the randomness of the donor’s appearance and the waiting time can be reasonably quantified and incorporated into the matching framework. Moreover, by discretizing the random continuous-time process into static decision stages, it can be proved that stable three-sided matching still exists in the case of random donor arrivals.

Based on the actual workflow of organ transplantation, the following queuing system is constructed:

Waiting time *Ek*: the waiting time of the organ recipient, i.e., the time until a suitable organ donor becomes available. This waiting time is characterized by randomness and non-Markovian properties, and thus can be modeled as following a *k*‑stage Erlang distribution with parameters *k* and λ.

Service time *M*: the duration from the start of the transplant surgery to its completion. Assuming that the efficiency of the medical team or equipment remains constant and that the service time is not influenced by previous surgeries (i.e., memoryless), the service time can be assumed to follow an exponential distribution.

Number of service counters *c*_*j*_: the number of simultaneous organ transplant surgeries that the *j*-th medical institution can handle.

System capacity limit *k*_*j*_: the maximum number of organ recipients that can be registered at the *j*-th medical institution.

Customer source size ∞: the total number of potential customers (recipients) who may arrive at medical institutions to receive service.

Service rule *PR*: organ recipients register their information at a medical institution and wait for a suitable donor organ to become available for transplantation. The medical institution determines priority for treatment based on the severity of the recipient’s condition and compatibility matching across relevant indicators. This process shares a structural similarity with the Priority Service (PS) mechanism in queuing theory.

**Assumption 1:** If all service counters are occupied when an organ recipient arrives, the recipient is willing to wait until a service becomes available.

**Assumption 2:** Each organ recipient in the queue has an equal probability of receiving priority treatment, i.e., the queue length is assumed to be 1.

When taking into account the waiting time cost of organ recipients at the *j*-th medical institution, the parameters of the Erlang distribution can be specified as *k* and $\lambda_{j}$. Here, *k* generally denotes the number of service stages required to treat a single organ recipient during the organ transplantation process, while $\lambda_{j}$ can represent the average service completion rate per unit time at the *j*-th medical institution, or the number of calls that the service desk can handle per unit time. For the sake of consistency, $\lambda_{j}$ is hereby defined as the number of transplant surgeries that can be performed simultaneously at the *j*-th medical institution, i.e., it is numerically equivalent to *c*_*j*_. Although the Erlang distribution itself does not possess the property of memory-lessness, the *k*‑stage Erlang distribution can be regarded as the distribution of the sum of *k* independent and identically distributed exponential random variables each with parameter λ. Therefore, the waiting time of each organ recipient at the *j*-th medical institution is given by *k*/$\lambda_{j}$ (*k*/*c*_*j*_, and *k* = 9, corresponding to the nine stages of organ transplantation: preliminary evaluation and consultation, tissue typing and assessment, document preparation and ethical review, surgical preparation, organ procurement and transplantation, postoperative monitoring and management, postoperative rehabilitation, follow-up and testing, as well as psychological support and recovery.)

**Fig 2:**
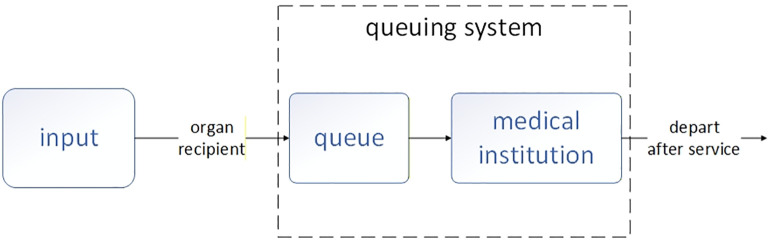
Queuing System for Organ Transplantation.

In summary, the organ transplantation process can be modeled as a queuing system $Ek/M/c_{j}/k_{j}/\infty /PR$, and the specific workflow is illustrated in Fig 2 below.

To prove the existence of a stable three-sided matching under such circumstances, the following explanation will be provided from the perspective of blocking triples in the mixed preference structure.

**Definition 3** ^[^28^]^ For a three-sided matching *M* with a hybrid preference structure, let the three preference orientations be denoted by A→B×C, $\vec{BA}$ and $\vec{CA}$ without loss of generality. If there exists a matching group$\left(a_{i},b_{j},c_{k}\right)\in \Omega -M$, such that the agents $a_{i},b_{j},c_{k}$ satisfy the following conditions respectively:

(1) For $a_{i}$, satisfy $\left(b_{j},c_{k}\right){≻}_{a_{i}}M_{A\to B\times C}\left(a_{i}\right)$;
(2) For $b_{j}$, satisfy $a_{i}{≻}_{b_{j}}M_{\vec{BA}}\left(b_{j}\right)$;
(3) For $c_{k}$, satisfy $a_{i}{≻}_{c_{k}}M_{\vec{CA}}\left(c_{k}\right)$;

Then the triple$\left(a_{i},b_{j},c_{k}\right)$is called a blocking triple of *M*.

**Theorem 1** Let $x_{ij}\;\;$ be a binary variable indicating whether individual *i* is matched with individual *j*匹配 (where *x*_*ij*_ = 1 denotes a match and *x*_*ij*_ = 0 denotes no match; the same applies to $y_{A\to B\times C}$ and $z_{ik}$). Let $\mu_{ij}$ denote the preference degree of individual *j* over individual *i*. A three-sided matching problem with a hybrid preference structure is stable if it satisfies the following conditions:


$$\begin{array}{c} \begin{array}{c} x_{ij}+\overset{i}{\underset{i^{*}:\mu_{i^{*}j}>\mu_{ij}}{\sum }}x_{i^{*}j}\geq 1\\ y_{A\to B\times C}+\overset{B\times C}{\underset{{\left(B\times C\right)}^{*}:\mu_{A\to {\left(B\times C\right)}^{*}}>\mu_{A\to B\times C}}{\sum }}y_{A\to {\left(B\times C\right)}^{*}}\geq 1\\ z_{ik}+\overset{i}{\underset{i^{*}:\mu_{i^{*}k}>\mu_{ik}}{\sum }}z_{i^{*}k}\geq 1 \end{array} \end{array}$$ (2)

**Proof:** Take the first constraint $x_{ij}+\overset{i}{\underset{i^{*}:\mu_{i^{*}j}>\mu_{ij}}{\sum }}x_{i^{*}j}\geq 1$ as an example.

Sufficiency:

Suppose there exists a blocking pair(*i*,*j*), i.e., *j* prefers *i* to its current matching partner *i*′, such that *x*_*ij*_ = 0. In this case, $\overset{i}{\underset{i^{*}:\mu_{i^{*}j}>\mu_{ij}}{\sum }}x_{i^{*}j}\geq 1$, meaning that there exists at least one agent $i^{*}$ satisfying $\mu_{i^{*}j}>\mu_{ij}$ and $x_{i^{*}j}=1$. That is, $i^{*}$ must be preferred to *i* by *j* within $\mu_{i^{*}j}>\mu_{ij}$, which contradicts the definition of the blocking pair (*i*,*j*). This completes the proof by contradiction.

Necessity:

Suppose there exists a stable matching *M*, but a matching pair (*i*,*j*) does not satisfy the constraint, i.e.,


$$\begin{array}{c} x_{ij}+\overset{i}{\underset{i^{*}:\mu_{i^{*}j}>\mu_{ij}}{\sum }}x_{i^{*}j}<1 \end{array}$$

This implies that *x*_*ij*_ = 0 and $\overset{i}{\underset{i^{*}:\mu_{i^{*}j}>\mu_{ij}}{\sum }}x_{i^{*}j}=0$. That is, the potential matching partner *i* of *j*, compared with the existing matching pair (*i*′,*j*), not only satisfies $\mu_{i^{\prime }j}<\mu_{ij}$, but also is not matched with *j*. In this case, (*i*,*j*) forms a blocking pair, which contradicts the definition of stability.

The proofs for $y_{A\to B\times C}+\overset{B\times C}{\underset{{\left(B\times C\right)}^{*}:\mu_{A\to {\left(B\times C\right)}^{*}}>\mu_{A\to B\times C}}{\sum }}y_{A\to {\left(B\times C\right)}^{*}}\geq 1$ and $z_{ik}+\overset{i}{\underset{i^{*}:\mu_{i^{*}k}>\mu_{ik}}{\sum }}z_{i^{*}k}\geq 1$ can be completed by following the same reasoning.

When the preference structure of a three-sided matching problem satisfies the hybrid preference structure, a stable matching can be constructed by virtue of the constraint conditions specified in Theorem 1.

**Theorem 2** There exists a stable matching in the queuing system $Ek/M/c_{j}/k_{j}/\infty /PR$ for the organ transplantation process.

Given that the arrival time of matching agents follows an Erlang distribution, the continuous-time process can be discretized into decision epochs (i.e., the instants when matching agents arrive). At each decision epoch, the system is reduced to a static three-sided matching problem.

Under static conditions, a three-sided matching problem with a hybrid preference structure is guaranteed to have a stable matching if no blocking triples are generated, and this result can be achieved by imposing the constraint conditions for stable matching specified in Theorem 1.

### 3.3. Preference misreporting and stability analysis

In the three-sided stable matching framework for organ transplantation, preference misreporting by organ recipients refers to the behavior in which recipients intentionally conceal or falsify their true health information—such as exaggerating illness severity or fabricating medical records—to obtain a higher matching priority and better access to organ resources. Such misrepresentation arises because, according to the impossibility theorem in stable matching theory(as shown in Theorem 3). In the context of scarce organ resources, recipients are incentivized to strategically misrepresent their conditions to gain advantages in waiting-list priority and medical institution selection, which would distort the true preference order among medical institutions, undermine the stability of matching outcomes, and lead to inefficient allocation and waste of medical resources. Therefore, it is necessary to analyze the impact of recipient misrepresentation and design a corresponding penalty mechanism to maintain the fairness and stability of the entire matching system.

**Theorem 3** [35]**(Impossibility Theorem)**There exists no stable matching mechanism under which truth-telling constitutes a dominant strategy for every participant(Dominant strategy: A strategy that yields an optimal response to any strategy adopted by other participants).

Theorem 3 indicates that a stable matching mechanism cannot simultaneously ensure that all participants report their true preferences. In the context of organ transplantation, recipients may misrepresent information—such as by falsifying medical records or exaggerating the severity of their conditions—to gain priority. This behavior will undermine the stability of matching results and lead to the waste of medical resources. We now prove the impact of such misrepresentation on the stability of matching outcomes during the matching process.

**Theorem 4** Misrepresentation behaviors of decision-makers during the matching process will exert an impact on the stability of matching outcomes.

**Proof:** Let *S* be the set of organ recipients and *R* be the set of medical institutions. For any two organ recipients s∈S and r∈R, there exists a priority treatment order determined by their own health conditions. Let ${≻}_{s}$ denote the preference order of organ recipient over medical institutions, and ${≻}_{r}$ denote the preference order of medical institution over organ recipients, respectively.

When a matching *M* is stable, there do not exist any organ recipient *s* and medical institution *r* such that $r{≻}_{s}M\left(s\right)$ and $s{≻}_{r}M\left(r\right)$, where M(s) denotes the hospital matched with organ recipient *s* under matching *M*, and M(r) denotes the organ recipient matched with medical institution *r* under matching *M*. Misrepresentation by organ recipient *s* refers to the act that *s* intentionally provides false information about his or her health status, which leads medical institution *r* to form a distorted preference ranking based on the falsified health status of *s*.

Suppose there exists a stable matching *M*, where each recipient *s* is matched with a corresponding institution *r* and all matching pairs satisfy the definition of stable matching. When recipient *s* engages in misrepresentation during the matching process—that is, *s* intentionally conceals his or her true health status—the preference ranking of institution *r* is thus distorted. Let ${{≻}_{r}}^{\prime }$ denote the true preference ranking of institution *r*, while the preference ranking expressed by *r* in the matching process is denoted by ${≻}_{r}$. Let ${{≻}_{s}}^{\prime }$ denote the true preference ranking of recipient *s* while the preference ranking declared by s in the matching process is denoted by ${≻}_{s}$, where ${{≻}_{r}}^{\prime }\neq {≻}_{r}$, ${{≻}_{s}}^{\prime }\neq {≻}_{s}$. Due to the misrepresentation of *s*, the matching result may be altered, yielding a new matching scheme denoted by $M^{\prime }$, where recipient *s* is matched with institution $r^{\prime }$, whereas *s*was originally matched with institution *r* under the stable matching *M.* The stability of the matching outcome after the occurrence of misrepresentation is discussed by cases as follows:

① If $r^{\prime }{{≻}^{\prime }}_{s}r$, i.e., *r*′ is preferred to *r* in the true preference ranking of *s*, then the misrepresentation of *s* may be motivated by the intention to receive treatment more quickly. However, this does not imply that *M*′ is stable, since *r*′ may have a more preferred matching partner $s^{\prime }$, i.e., $s^{\prime }{≻}_{r^{\prime }}s$.

② If $r{{≻}^{\prime }}_{s}r^{\prime }$, i.e., *r* is preferred to *r*′ in the true preference ranking of *s*, then the misrepresentation of *s* may stem from other motivations. In this case, *s* will end up with a less satisfactory matching partner due to misrepresentation, while undermining the original stable matching *M*.

Therefore, for $M^{\prime }$ to be stable, it is a necessary condition that for all *r* and *s*, there does not exist a case where $r{≻}_{s}M^{\prime }\left(s\right)$ and $s{≻}_{r}M^{\prime }\left(r\right)$. However, misrepresentation by s may alter the preference relation between r and s, thereby undermining the stability of the matching.

According to the proofs elaborated above, in the context of three-sided matching for organ transplantation, misrepresentation by organ donors may impair the stability of matching outcomes. To mitigate the medical risks arising from such scenarios, medical institutions and organ recipients can, in addition to strengthening doctor-patient communication, refining medical records and enhancing medical ethics education, formulate corresponding penalty mechanisms (e.g., lowering matching priority) during the matching process. Therefore, a penalty operator is designed hereinafter to reduce the inaccuracies or instabilities in matching outcomes caused by misrepresentation or concealment of information. Let $I_{i},{I^{\prime }}_{i}\in \left(0,1\right)$ denote the severity of the true medical condition and the condition self-reported by the patient, respectively. A penalty operator $S_{i}=\lambda \left|I_{i}-{I^{\prime }}_{i}\right|$ is designed based on the discrepancy between the recipient’s self-reported condition and the true medical condition, where λ∈[0,1] is the penalty coefficient.

## 4. Model construction

**Fig 3:**
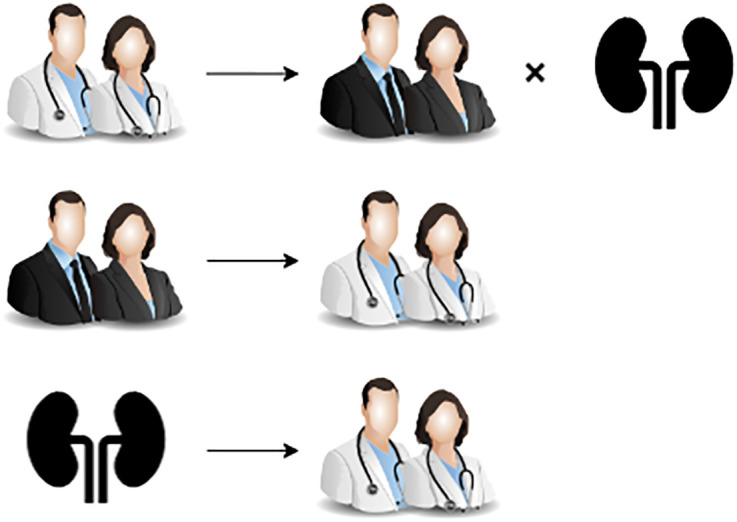
Schematic Diagram of Three-sided Matching Preferences.

As can be concluded from the above discussion, the three-sided matching problem in the context of organ transplantation possesses the stability property, and its preference structure is illustrated in Fig 3. We now formally describe this problem as follows: there are *i* organ recipients selecting and registering with *j* medical institutions, and *k* organ donors waiting to treat the recipients via these medical institutions. Specifically, each organ recipient can only be matched with one organ donor and one medical institution, and each organ donor can also only be matched with one organ recipient and one medical institution. Given the mutual evaluations among organ donors, organ recipients, and medical institutions, the core question to be addressed is how to conduct the matching to maximize the matching efficiency.

Let i={1,2,⋯,m} be the set of organ recipients, j={1,2,⋯,n} be the set of medical institutions, and k={1,2,⋯,p} be the set of organ donors. Let *C*_*ij*_ denote the treatment cost incurred when organ recipient *i* seeks medical treatment at medical institution *j*, where *C*_*ij*_ consists of the medical cost *C*_*j*_ and the time cost *L*_*ij*_. Let $M_{ij},M_{jk}$ represent the evaluations of the medical level of institution *j* given by organ recipient *i* and organ donor *k*, respectively. Let *I*_*i*_ denote the severity of the medical condition of recipient *i*, and *O*_*i*_ denote the postoperative recovery status of recipient *i*. Let $T_{ik}\in \left[0,1\right]$ denote the matching success rate between organ recipient *i* and organ donor *k*. Let *x*_*ij*_ be a 0–1 variable indicating whether organ recipient *i* is matched with medical institution *j*; let *y*_*jk*_ be a 0–1 variable indicating whether medical institution *j* is matched with organ donor *k*; let *z*_*ik*_ be a 0–1 variable indicating whether organ recipient *i* is matched with organ donor *k*. Let *μ*_*ijk*_ be a 0–1 variable representing whether organ donor *k* is willing to treat organ recipient *i* with the assistance of medical institution *j*. Specifically, all these variables take the value of 1 if the corresponding matching is successful, and 0 otherwise. Let $\mu_{ijk}=x_{ij}\cdot y_{jk}\cdot z_{ik}$ denote a three-sided matching tuple.

Based on the comprehensive analysis above, the three-sided stable matching model for organ transplantation can be constructed as follows:


$$\begin{array}{c} max\;f_{\text{1}}=\sum_{i=1}^{m}\sum_{j=1}^{n}\left(C_{ij}+M_{ij}\right)\cdot x_{ij} \end{array}$$ (3)

$$\begin{array}{c} max\;f_{\text{2}}=z_{ik}\cdot T_{ik}\cdot \sum_{i=1}^{m}\sum_{j=1}^{n}\sum_{k=1}^{p}\left[\mu_{ijk}\cdot \left(I_{i}\text{+}O_{i}\right)-S_{i}\right] \end{array}$$ (4)

$$\begin{array}{c} max\;f_{\text{3}}=\sum_{j=1}^{n}\sum_{k=1}^{p}\left(M_{jk}\cdot y_{jk}\right) \end{array}$$ (5)

$$\begin{array}{c} \sum_{j=1}^{j}x_{ij}\leq 1 \end{array}$$ (6)

$$\begin{array}{c} \sum_{j=1}^{j}y_{jk}\leq 1 \end{array}$$ (7)

$$\begin{array}{c} \sum_{i=1}^{i}z_{ik}\leq 1 \end{array}$$ (8)

$$\begin{array}{c} \sum_{k=1}^{k}z_{ik}\leq 1 \end{array}$$ (9)

$$\begin{array}{c} \left\{ \begin{array}{c} @l@T_{ik}\geq 0.6,z_{ik}=1\\ T_{ik}\leq 0.6,z_{ik}=0 \end{array}\right. \end{array}$$ (10)

$$\begin{array}{c} x_{ij}+\overset{i}{\underset{i^{*}:\mu_{i^{*}j}>\mu_{ij}}{\sum }}x_{i^{*}j}\geq 1 \end{array}$$ (11)

$$\begin{array}{c} y_{A\to B\times C}+\overset{B\times C}{\underset{{\left(B\times C\right)}^{*}:\mu_{A\to {\left(B\times C\right)}^{*}}>\mu_{A\to B\times C}}{\sum }}y_{A\to {\left(B\times C\right)}^{*}}\geq 1 \end{array}$$ (12)

$$\begin{array}{c} z_{ik}+\overset{i}{\underset{i^{*}:\mu_{i^{*}k}>\mu_{ik}}{\sum }}z_{i^{*}k}\geq 1 \end{array}$$ (13)

$$\begin{array}{c} x_{ij},y_{jk},z_{ik},\mu_{ijk}=\left\{ \begin{array}{c} @l@1,successful\;\;matching\\ 0,unsuccessful\;\;matching \end{array}\right. \end{array}$$ (14)

Equation represents the overall satisfaction degree of organ recipients with respect to medical institutions; Equation represents the overall satisfaction degree of medical institutions with respect to organ recipients and organ donors, where $S_{i}=\lambda \left|I_{i}-{I^{\prime }}_{i}\right|$ denotes the penalty function (with λ∈[0,1]as the penalty coefficient, and $I_{i},{I^{\prime }}_{i}\in \left(0,1\right)$denoting the severity of the true medical condition and the condition self-reported by the patient, respectively); Equation represents the overall satisfaction degree of organ donors with respect to medical institutions; Equation restricts that each organ recipient can be matched with at most one medical institution; Equation restricts that each organ donor can be matched with at most one medical institution; Equation restricts that each organ recipient can be matched with at most one organ donor; Equation restricts that each organ donor can be matched with at most one organ recipient; Equation imposes constraints on the matching success rate between organ recipients and organ donors; Equations, equations and equations restrict the stable matching conditions under the hybrid preference structure; Equation restricts that a matching tuple can only be formed if all three matching parties are voluntary and the matching complies with medical principles.

For the obtained solution set, a weighted calculation can be performed for each objective according to the actual preferences, namely:


$$\begin{array}{c} max\;F=W_{\text{1}}f_{\text{1}}+W_{\text{2}}f_{\text{2}}+W_{\text{3}}f_{\text{3}} \end{array}$$ (15)

The objectives are integrated to obtain the optimal stable matching, where:


$$\begin{array}{c} W_{\text{1}},W_{\text{2}},W_{\text{3}}>0 \end{array}$$ (16)

$$\begin{array}{c} W_{\text{1}}+W_{\text{2}}+W_{\text{3}}=1 \end{array}$$ (17)

$$\begin{array}{c} f_{\text{1}}\cdot f_{\text{2}}\cdot f_{\text{3}}>0 \end{array}$$ (18)

Moreover, the Equation ensures that the optimality of the solution is not achieved at the expense of reducing any objective value to 0.

For the cost indicators (i.e., medical cost and time cost), dimensionless inverse transformation can be performed, namely, $x^{\prime }=(x_{max}-x)/(x_{max}-x_{min})$ for each indicator. For all other indicators, **dimensionless positive transformation** can be conducted, namely, $x^{\prime }=(x-x_{min})/(x_{max}-x_{min})$, for each indicator. These transformations compress the value range of all indicators into the interval [0,1].

## 5. Improved NSGA-III solution algorithm

### 5.1. Improved Design of NSGA-III Algorithm

The three-sided matching model for organ transplantation is characterized by two core features: multi-objective optimization and large-scale problem solving.

(1) It is necessary to coordinate multiple potentially conflicting objectives to obtain the Pareto optimal solution set;
(2) Traditional deterministic algorithms face the problem of exponential explosion of computational complexity.

Based on the improved intelligent optimization algorithm, an efficient solution approach is provided for large-scale matching problems [36]. Compared with the NSGA-II algorithm, the NSGA-III algorithm can not only solve more complex multi-objective programming problems, but also introduce the strategies of reference point selection and the concept of hypercube division on the basis of NSGA-II. These improvements enhance the convergence and search efficiency of the algorithm while maintaining the diversity of solutions.The improvement of NSGA-III mainly focuses on how to further preserve solution diversity and avoid the algorithm falling into local optima. The integration with the bird swarm algorithm (BSA) can enhance the search capability of NSGA-III. Therefore, an improved NSGA-III algorithm is proposed to solve the model. This algorithm can not only obtain the Pareto optimal solutions, but also reduce the computational scale, thus exhibiting stronger search ability and diversity maintenance capability.

(1) Initialization Phase

The Logistic chaotic mapping initialization strategy of the Bird Swarm Algorithm (BSA) is adopted to randomly generate a set of initial solutions, which serve as the initial population of the algorithm to ensure the diversity and distribution of the population. Its mathematical expression is as follows:


$$\begin{array}{c} x_{i+1}=ax_{i}\left(1-x_{i}\right) \end{array}$$ (19)

where *a* is the control parameter, whose value is selected from the interval (0,.4]. The larger the value of *a*, the stronger the chaotic behavior; when *a* = 4, the system is in a state of complete chaos.

(2) Update and Evolution Phase

①The position update formulas of the Bird Swarm Algorithm (BSA) are adopted to simulate the flight behavior and foraging behavior of birds during the search process, and each individual is updated accordingly. In the flight behavior, individuals are divided into producers and scroungers based on their fitness levels. The position update formula for producers is as follows:


$$\begin{array}{c} \overset{t+1}{\underset{i}{x}}\;=\overset{t}{\underset{i}{x}}\;+\overset{t}{\underset{i}{x}}\;\cdot randn\left(0,1\right) \end{array}$$ (20)

The position update formula for scroungers is as follows:


$$\begin{array}{c} x\;it+1=\overset{t}{\underset{i}{x}}\;+(x\;kt-x\;it)\cdot FL\cdot rand\left(0,1\right) \end{array}$$ (21)

Where *randn*(0,1) represents a random number generated following a Gaussian distribution with a mean of 0 and a standard deviation of 1, and k∈[1,N] and k≠i, where FL∈[0,1] denotes the probability that scroungers forage along with producers. The position update formula for individuals in foraging behavior is as follows:


$$\begin{array}{c} x\;it+1=x\;it+(p_{i}\;-\overset{t}{\underset{i}{x}}\;)\cdot C\cdot rand\left(0,1\right)+(g_{j}-\overset{t}{\underset{i}{x}}\;)\cdot S\cdot rand\left(0,1\right) \end{array}$$ (22)

$\overset{t}{\underset{i}{x}}$ denotes the current position of the individual, $p_{i}$ denotes the optimal position traversed by the *i*-th individual, and $g_{j}$ denotes the global optimal position of the population. *C* and *S* are two positive constants, referred to as the cognitive coefficient and the social evolution coefficient, respectively.

②Crossover and mutation operations are performed on the population to generate a new offspring population. The specific operation steps are as follows:

Crossover positions and a random number r∈[0,1] are generated randomly: if $r>p_{c}$ (where $p_{c}\in \left(0,1\right)$ denotes the given crossover probability), the parent individuals are copied as the crossover results; if $r\leq p_{c}$, a single-point crossover operation is performed: a crossover point is randomly selected in the encoding string of the individuals, and then the genetic information of the two parent individuals after the crossover point is exchanged to generate two new offspring individuals.

Mutation positions, mutation values, and a random number r∈[0,1] (where *r* follows a uniform distribution) are generated randomly: if $r<p_{m}$ (where $p_{m}\in \left(0,1\right)$ denotes the given mutation probability), the parent individuals are copied as the mutation results; if $r\geq p_{m}$, considering that the traditional fixed-step mutation operator is difficult to balance both global search and local optimization performance, and the perturbation amplitude required by the population in different regions of the solution space varies significantly. To enable the mutation step size to adaptively change according to the individual’s position, a larger perturbation is achieved in sparse regions to enhance exploration, and a smaller perturbation is implemented in dense regions to improve convergence. This part combines the nonlinear, monotonically increasing, and gradually changing characteristics of a function to introduce the square root structure $\sqrt{x_{i}}$ of variable *x*_*i*_ to construct an adaptive mutation operator. The square root function has the characteristic of amplifying small values and compressing large values, which can match the distribution characteristics of the population, thus being able to meet the dynamic adjustment requirements of the adaptive step size. Based on the above idea, the improved mutation operation as shown in Equation (23) is designed:


$$\begin{array}{c} x_{i+1}=\frac{\sqrt{x_{i}}+r}{\text{2}} \end{array}$$ (23)

**Note:** Next, the solution performance of the improved mutation rule will be verified from three aspects: ergodicity, local search capability, and global search capability.

a Ergodicity

State Space: The feasible solution space $\chi \subseteq {\mathbb{R}}^{n}$;

State Transition Probability: Given the current state *x*_*i*_, the probability density of the transition $P\left(x_{i+1}|x_{i}\;\right)$ to the next state *x*_*i*+1_is defined as:


$$\begin{array}{c} P\left(x_{i+1}|x_{i}\;\right)=2x_{i+1}\;-\sqrt{x_{i}} \end{array}$$ (24)

Due to the randomness of *r*, *x*_*i*+1_ can cover the interval [0,1] through multiple iterations. Therefore, for any two arbitrary states $x_{a},x_{b}\in \chi$, there exists a finite number of mutation operations that enable the transition from state *x*_*a*_ to state *x*_*b*_；

Since *r* is a continuous variable, the recurrence time has no fixed period. Therefore, there does not exist a fixed period *k* such that *x*_*i*_ can only return to itself every *k* steps.

In summary, the NSGA-III algorithm with the improved mutation rule defined in Equation has ergodicity.

b Local Search Capability

It is not difficult to observe that equation is monotonic, bounded above, and has an extreme value. Let the equilibrium point $x^{*}$ be a solution that no longer changes under the mutation operation, i.e., it satisfies:


$$\begin{array}{c} x^{*}=\frac{\sqrt{x^{*}}+r}{\text{2}} \end{array}$$ (25)

It follows that $x^{*}={\left(\frac{1+\sqrt{1+8r}}{\text{4}}\right)}^{\text{2}}$.

Let $x_{i+1}=f\left(x_{i}\right)=\frac{\sqrt{x_{i}}+r}{\text{2}}$, then $f^{\prime }\left(x\right)=\frac{\text{1}}{4\sqrt{x}}$, and $f^{\prime }\left(x\right)$ is continuous on the interval (0,1]. Substituting the equilibrium point $x^{\prime }$into the function yields $f^{\prime }\left(x^{*}\right)=\frac{\text{1}}{1+\sqrt{1+8r}}$. According to the description of local convergence at the equilibrium point by Li Qingyang et al*.*[37], since $\left|f^{\prime }\left(x^{*}\right)\right|<1$, the algorithm is locally convergent at the equilibrium point $x^{*}$ for all values of *r*.

c Global Search Capability

For small values of *x*, when $x\to {\text{0}}^{+}$, $f^{\prime }\left(x\right)\to +\infty$. Thus, in the edge or low-density regions of the solution space, minor changes are significantly amplified, further forcing the population to explore unknown regions.

For large values of *x*, when x→1, $f^{\prime }\left(x\right)\to \frac{\text{1}}{\text{4}}$. The rate of change decreases, which means that in regions close to the optimal solution, the mutation step size is automatically reduced to achieve refined search.

Based on the theoretical proofs from the above aspects, it can be concluded that the improved mutation rule has good feasibility.

③The parent population and offspring population are merged, and non-dominated sorting is performed on the combined population.

(3) Selection and Retention Phase

①According to the reference point mechanism of NSGA-III, the distance between each individual and the reference points is calculated, and the quality of these individuals is evaluated. Assume that the number of optimization objectives is *M* and each optimization objective is divided into *p* equal parts; the calculation formula for the number of reference points *H* is as follows:


$$\begin{array}{c} H=\overset{M+p-1}{\underset{p}{C}} \end{array}$$ (26)

To prevent the algorithm from getting stuck in local optimal convergence, the target space is uniformly divided based on the number of reference points, resulting in several subspaces. In each subspace, the median point is selected as the reference point, and the crowding distance from each individual to each reference point is calculated. The larger the crowding distance, the more uniform the distribution of the population in the target space and the better the diversity, thereby effectively suppressing the occurrence of local convergence in the algorithm.

②The elite retention strategy is adopted to directly retain the optimal solutions in the current population into the next generation, thus avoiding the loss of excellent solutions.

(4) Iteration and Evolution Phase

### 5.2. Specific Implementation Steps of the Algorithm

The pseudo-code for the specific implementation steps is as follows:

Improved NSGA-III Solution Algorithm

1: **Input:** Crossover probability *p*_*c*_, Mutation probability *p*_*m*_, Population size *N*, Maximum iterations *T*_*max*_, Control parameter *a* = 4

2: **Output:** Optimal three-sided stable matching scheme

3: **Step 1**: Initialization

4: **For** *i* = 1 to n **do**

5:   $x_{i+1}=ax_{i}\left(1-x_{i}\right)$

6: **End for**

7: Generate reference points for NSGA-III

8: **Step 2**: The stage of update

9:  **For** Each Individual in Population **do**

10:   **If** Fitness(Individual) is High **do**

11:    $\overset{t+1}{\underset{i}{x}}\;=\overset{t}{\underset{i}{x}}\;+\overset{t}{\underset{i}{x}}\;\cdot randn\left(0,1\right)$

12:    **Else do**

13:     $x\;it+1=x\;it+(p_{i}\;-\overset{t}{\underset{i}{x}}\;)\cdot C\cdot rand\left(0,1\right)+(g_{j}-\overset{t}{\underset{i}{x}}\;)\cdot S\cdot rand\left(0,1\right)$

14:     **End if**

15:   **End for**

16: **while** *t* ≤ *T*_*max*_ **do**

17: **Step3**: evolution stage

18: **For** *x*_*i*_ = 1 to *n* **do**

19:   **If** $r>p_{c}$ **do**

20:    *X*_*i* + 1_ = *x*_*i*_

21:   **Else do**

22:    Single-point crossover operation

23:   **End if**

24: **End for**

25: **For** *x*_*i*_ = 1 to *n* **do**

26:   **If** $r<p_{m}$ **do**

27:    *X*_*i* + 1_ = *x*_*i*_

28:   **Else do**

29:     $x_{i+1}=\frac{\sqrt{x_{i}}+r}{\text{2}}$

30:  **End if**

31: **End for**

32: Form final offspring population *Q*

33: **Step4**: Merge the parent and offspring populations and perform non-dominated sorting

34: Merge parent population *P* and offspring population *Q* into combined population *R*

35: Conduct non-dominated sorting on *R*

36: **Step5**: Selection and Retention Phase

37: **Step6**: Update iteration counter *t* = *t* + 1

38: **End while**

## 6. Case analysis

### 6.1. Experiments and results

According to the latest 2023 data from the *China Organ Transplant Response System* (COTRS), the number of people registered and waiting for organ transplants in China has exceeded 140,000, while the number of patients receiving organ transplants each year is less than 20,000. Based on the relevant data in 2023: there are approximately 180 medical institutions qualified to perform organ transplant surgeries; the number of organ donors who registered and successfully completed donations in that year was about 6,450; the annual average number of patients registered and waiting for transplants is around 100,000. In this experiment, simulated data is constructed in accordance with the actual ratio of the quantities of the three parties involved in the above data.

A total of 10 medical institutions with the necessary qualifications for organ transplantation surgeries, 358 registered organ donors, and 5555 registered organ recipients who were waiting for organ transplantation were selected as the research subjects. The data inclusion criteria were that the donor’s organ function was qualified, the recipient’s condition assessment was complete, and the matching basic indicators met the standards. Samples with major underlying diseases that were contraindicated or not in line with medical ethics were excluded. In the data preprocessing stage, uncertain indicators such as medical level, severity of the disease, and postoperative recovery expectations were quantified using intuitive fuzzy information. A scoring function was constructed using membership degrees, non-membership degrees, and hesitation degrees to complete the preference ranking. A 9th-order Erlang distribution was used to depict the random characteristics of the donor’s arrival and the non-Markov characteristics of the recipient’s waiting time. At the same time, the possible false reporting of the recipient’s condition was simulated, and a penalty coefficient suppression strategy was set to correct the distortion(where the penalty coefficient λ=0.5). The cost indicators were inverted and positively transformed, and the benefit indicators were linearly normalized. Missing values were filled using the zero-padding strategy. The data set as a whole satisfied the clinical matching logic for organ transplantation and the mixed preference structure of the three-sided matching.

**Fig 4:**
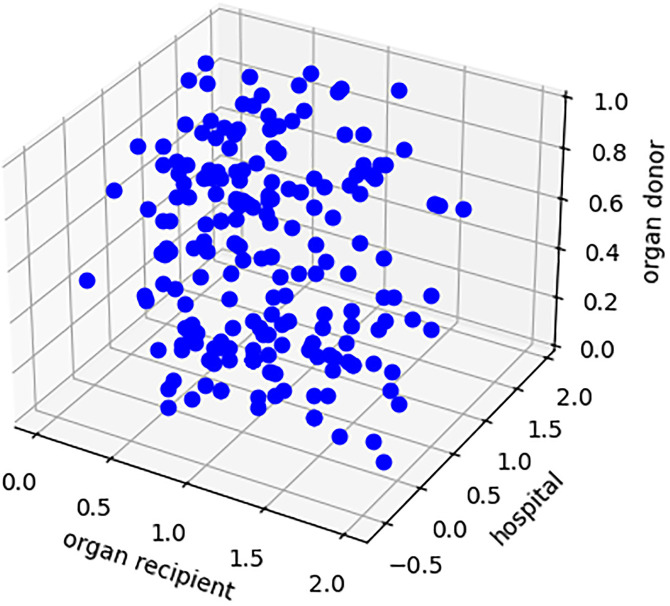
Initial Population.

**Fig 5:**
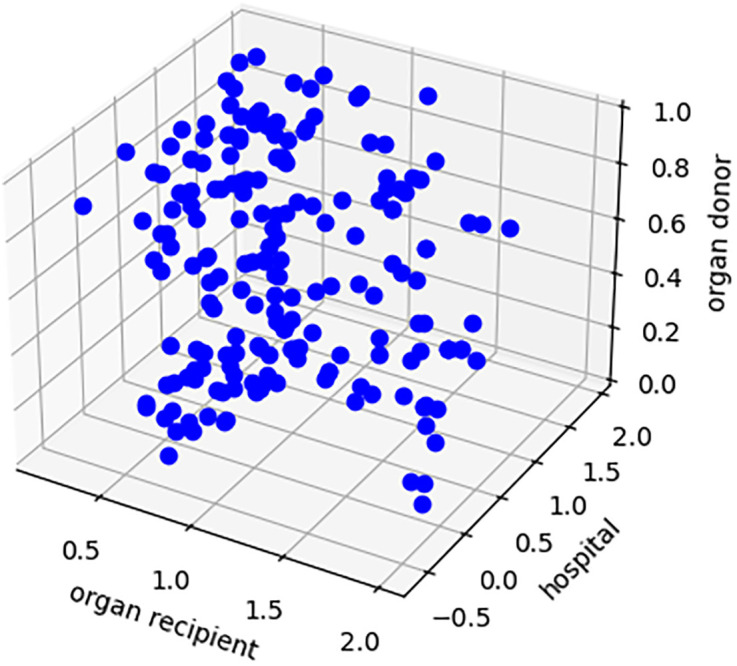
Population after Crossover and Mutation.

**Fig 6:**
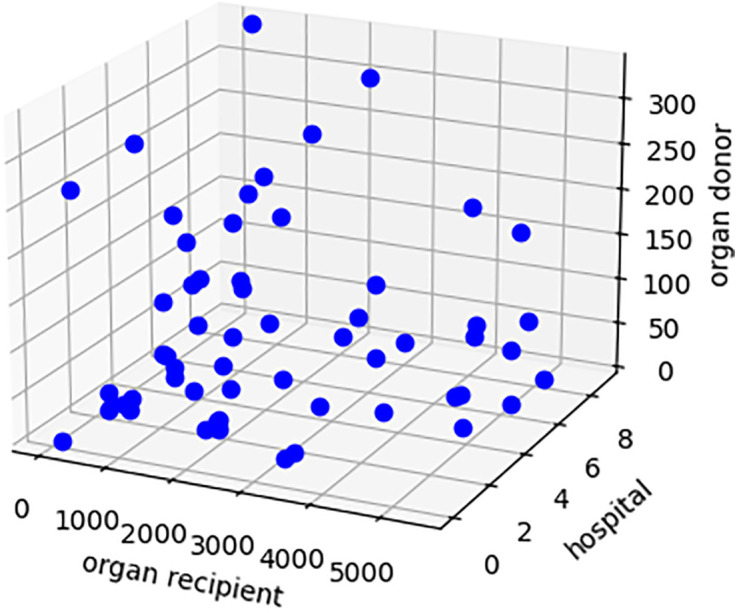
Experimental Results of Three-sided Matching.

The crossover probability was set to 0.90, the mutation probability to 0.05, the population size to 60, and the number of iterations to 10. *FL* = 0.5, *C* = 0.5, *S*=0.2. The weights were set as $w_{\text{1}},w_{\text{2}},w_{\text{3}}=$(0.3,0.5,0.2). in accordance with the requirements of the actual background. The experiment was conducted on a computer equipped with a 12th Gen Intel(R) Core(TM) i5-12500H 2.50 GHz processor and 16.0 GB of RAM, with Python 3.10.11 used as the auxiliary computing software. To intuitively reflect the change in the number of feasible solutions after each genetic operation, Fig 4 and Fig 5 is presented below (the dots in the figure represent the satisfaction values of the three-sided matching subjects under the corresponding matching pairs), and the final matching results are shown in Fig 6 (the dots in the figure represent the corresponding numbers of each element in the subject set under the matching pairs):

As shown in Figs 4–6, the improved NSGA-III algorithm not only finds multiple non-dominated solutions within a limited number of iterations, providing a variety of feasible solutions for the organ allocation problem in organ transplant surgeries, but also generates 58 sets of stable three-sided matches among organ recipients, medical institutions, and organ donors in this experiment. This number is far smaller than the number of registered patients waiting for treatment, and there also exists the problem of uneven resource allocation among different medical institutions. From a practical perspective, the experimental results are basically consistent with the existing problems faced by China’s current organ transplantation field.

**Fig 7:**
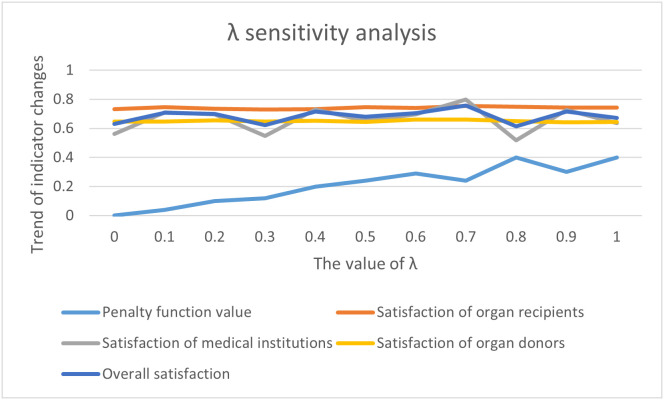
λ Sensitivity analysis.

In the three-sided matching model for organ transplantation based on multi-objective optimization, the penalty coefficient *λ* is the core parameter that regulates the severity of punishment for the liar’s behavior by the recipient. Its value directly affects the effectiveness of the penalty function, hospital satisfaction, and the effectiveness of the matching. To verify the degree of influence of *λ* on the model output, identify the sensitive range and the optimal value range, this study conducts a sensitivity analysis within the λ∈[0,1] interval to provide a quantitative basis for the practical application of the model:

The core test variable is the penalty coefficient *λ*, which takes values within the range of [0,1] and is uniformly selected at intervals of 0.1. The specific test set is. Other parameters remain unchanged. The core observation indicators are: the penalty function value and the satisfaction of the three parties involved in the matching. The test results are shown in Fig 7:

Fig 7 presents the sensitivity analysis results of the penalty coefficient *λ* on the penalty function value and the satisfaction of the three parties. The data graph shows that the penalty function value monotonically increases as *λ* increases, reaching a peak at *λ* = 1; the satisfaction of the organ recipients fluctuates slightly overall and stabilizes within the range of 0.73–0.75, achieving the optimal value at *λ* = 0.7; the satisfaction of the medical institutions is the most sensitive to *λ*, showing a significant rising and then falling trend, reaching the highest point at *λ* = 0.7; while the satisfaction of the organ donors is almost unaffected by the change of *λ*, remaining at a stable level over the long term. The combined effect of the satisfaction of the three parties leads to a U-shaped inverted characteristic of the total satisfaction with respect to *λ* variation, reaching the global optimal value at *λ* = 0.7, while the situations of *λ* = 0 (no punishment) and *λ* ≥ 0.8 (excessive punishment) significantly lower the overall performance. The regulatory effect of *λ* essentially reshapes the information authenticity through the penalty function: moderate punishment (*λ* = 0.6–0.7) effectively inhibits false reporting behavior, improving the authenticity and satisfaction of the hospital preference ranking; excessive punishment reduces the feasible matching space and damages the medical experience; no punishment leads to information disorder and causes the total satisfaction to drop to the lowest point. This result deeply confirms the core supporting role of integrity constraints in the organ transplantation matching system, confirming the necessity of institutionalized punishment for maintaining stable matching, precisely locating *λ* ≈ 0.7 as the optimal equilibrium point that balances integrity governance and the experiences of the three parties, and providing a quantitative basis for formulating scientific allocation rules in practice.

To verify the solution performance of the improved NSGA-III algorithm, the following verification will be conducted from the aspects of algorithm complexity, effectiveness, and ablation experiments.

### 6.2. Algorithm complexity analysis

To further analyze the solution performance of the proposed improved NSGA-III algorithm, several modified optimization algorithms based on NSGA-III proposed in recent years as well as the original NSGA-III algorithm are listed below. The performance of these different algorithms is analyzed using the indicator of algorithm complexity. As can be seen from Table 1 below, the improved NSGA-III algorithm does not show a significant increase in algorithm complexity. However, the integration of the Bird Swarm Algorithm (BSA) has greatly improved the computational accuracy and result diversity of the entire new hybrid optimization algorithm. It breaks through the dilemma of premature convergence or local convergence of the traditional NSGA-III algorithm, thus endowing the algorithm with better feasibility.

**Table 1:** Complexity Comparison of Different Improved Algorithms Based on NSGA-III.

Algorithm	Time Complexity	Notation Explanation
NSGA-III [38]	$O\left(N^{\text{2}}M\right)$	*N* denotes the population size, *M* denotes the number of objective functions
NSGAIII-OSTWS [39]	Max(O(DN),O(mN|W|))	*D* denotes the number of decision variables, *N* denotes the population size, *m* denotes the number of objective functions, |W|denotes the length of the reference vector *W*
MOITGO [40]	$\begin{array}{c} O\left(MN^{\text{2}}\right)+\\ O\left(\left(kN+n\right)\left(K+M\right)\right) \end{array}$	*N* denotes the population size, *M* denotes the number of objective functions, *K* denotes the number of genes in a chromosome, *n* denotes the number of cell mutation events, *k* denotes the cell growth cycle
Improved NSGA-III Algorithm	$O\left(T\left(N^{\text{2}}+NM\right)\right)$	*T* denotes the number of iterations, *N* denotes the population size, *M* denotes the number of objective functions

### 6.3. Algorithm effectiveness analysis

Two metrics, IGD [41](Inverted Generational Distance) and NPS [42](Number of Pareto Solutions), are adopted to measure the convergence and diversity of the algorithms, respectively. To verify the solution performance of the improved NSGA-III algorithm, comparative tests were conducted on the improved NSGA-III algorithm, the original NSGA-III algorithm, and the NSGA-II algorithm across test suites with different scales and optimization criteria (including ZDT1, ZDT2, DTLZ1, WFG1, and LSOMP1). The corresponding IGD and NPS values were obtained. Uniform parameters were set for the experiments: the number of iterations was 50, the population size was 100, the number of decision variables was 30, and the number of reference points was 100. All algorithms were independently run ten times, with the average values taken. The experimental results are presented in Table 2 below:

**Table 2:** Comparison of Solution Performance of the Improved NSGA-III Algorithm.

	Improved NSGA-III Algorithm		NSGA-III		NSGA-II	
	IGD	NPS	IGD	NPS	IGD	NPS
ZDT1	0.24	68	1.37	41	0.29	63
ZDT2	0.47	27	0.76	25	0.65	23
DTLZ1	1.79	98	2.09	97	2.55	72
WFG1	0.20	83	0.17	79	0.34	81
LSOMP1	0.36	41	1.35	35	0.67	29

(1) Convergence (IGD)

On most test problems, the improved NSGA-III algorithm yields lower IGD values, indicating its superior convergence performance. For instance, on the ZDT1, ZDT2, DTLZ1, and LSOMP1 problems, the IGD values of the improved NSGA-III algorithm are all lower than those of the NSGA-III and NSGA-II algorithms. Particularly on the ZDT1 and ZDT2 problems, the IGD values of the improved NSGA-III algorithm are significantly lower than those of the other two algorithms.

On the WFG1 problem, the IGD value of the improved NSGA-III algorithm is slightly higher than that of the NSGA-III algorithm. The main reasons may be as follows: ① The WFG1 test suite itself has high complexity, which tends to generate unstable results; ② The algorithm parameter settings may not be applicable to the context of the WFG1 test suite.

(2) Diversity (NPS)

On most test problems, the improved NSGA-III algorithm achieves higher NPS values, indicating its excellent diversity performance. For instance, on the ZDT1, DTLZ1, and WFG1 problems, the NPS values of the improved NSGA-III algorithm are significantly higher than those of the NSGA-III and NSGA-II algorithms. Particularly on the ZDT1 and DTLZ1 problems, the NPS values of the improved NSGA-III algorithm are obviously superior to those of the other two algorithms.

In summary, the improved NSGA-III algorithm exhibits favorable performance in both convergence and diversity across most test problems.

In order to evaluate the performance of the proposed improved NSGA-III algorithm in multi-objective optimization problems, this section conducts pairwise statistical significance analysis using the Wilcoxon signed-rank test under the test environment in Table 2 (carry out 30 independent runs, and additionally incorporate the comparison of the greedy algorithm and random search), with the significance level set at 0.05. Table 3 summarizes the statistical test results of the improved NSGA-III and the comparison algorithms on each test problem. The *p*-value reflects the significance of the performance difference, and if p<0.05, it indicates that the difference is statistically significant. The significance test results are as shown in Table 3:

**Table 3:** Wilcoxon Signed-Rank Test.

Test question	Control group	*p*-value	Significance
ZDT1	Improved NSGA-III vs NSGA-II	0.0216	Remarkable
	Improved NSGA-III vs NSGA-III	0.0089	Remarkable
	Improved NSGA-III vs Greedy	0.0293	Remarkable
	Improved NSGA-III vs Random	0.0076	Remarkable
ZDT2	Improved NSGA-III vs NSGA-II	0.0312	Remarkable
	Improved NSGA-III vs NSGA-III	0.0427	Remarkable
	Improved NSGA-III vs Greedy	0.0043	Remarkable
	Improved NSGA-III vs Random	0.0012	Remarkable
DTLZ1	Improved NSGA-III vs NSGA-II	0.0189	Remarkable
	Improved NSGA-III vs NSGA-III	0.0356	Remarkable
	Improved NSGA-III vs Greedy	0.0276	Remarkable
	Improved NSGA-III vs Random	0.0007	Remarkable
WFG1	Improved NSGA-III vs NSGA-II	0.000002	Remarkable
	Improved NSGA-III vs NSGA-III	0.000002	Remarkable
	Improved NSGA-III vs Greedy	0.0165	Remarkable
	Improved NSGA-III vs Random	0.0172	Remarkable
LSOMP1	Improved NSGA-III vs NSGA-II	0.048104	Remarkable
	Improved NSGA-III vs NSGA-III	0.0491	Remarkable
	Improved NSGA-III vs Greedy	0.0384	Remarkable
	Improved NSGA-III vs Random	0.0128	Remarkable

From Table 3, it can be seen that the improved NSGA-III achieved statistically significant differences (p<0.05) in all 20 comparison groups. Specifically, in the WFG1 test problem, the p-values of the improved algorithm, NSGA-II, and NSGA-III were all 0.000002, reaching an extremely significant level. On ZDT1, ZDT2, DTLZ1, and LSOMP1, all p-values ranged from 0.0007 to 0.0491, meeting the significance criteria. Moreover, the improved NSGA-III also outperformed the pure greedy algorithm and random search in all test problems.

The above statistical results indicate that combining the initialization strategy of the bird swarm algorithm and the adaptive mutation operation enables the improved NSGA-III to achieve better results in terms of convergence and diversity compared to the comparison algorithms, which verifies the effectiveness of the proposed improvement strategy.

### 6.4. Ablation experiments

To further verify the effectiveness of each improved component in the algorithm, a systematic analysis was conducted via Ablation Study. The Hypervolume metric (HV) was adopted as the evaluation criterion to simultaneously measure the convergence and distribution of the solution set, where a higher value indicates better algorithm performance. Meanwhile, stability (Std HV) was introduced to reflect the fluctuation degree of HV values during multiple independent runs of the algorithm. A lower Std HV value implies a weaker impact of different components (PSO initialization, improved mutation) on the stability of the algorithm. The test function was DTLZ1 (with 20 experimental runs), and the specific experimental design and results are presented in Table 4 below:

**Table 4:** Results of Ablation Experiments.

Configuration Name	Initialization Method	Mutation Operator	Design Characteristics	Average HV Performance	Stability (Std HV)
Improved NSGA-III Algorithm	PSO Initialization	Improved Mutation Rule	Full Algorithm with All Improved Components	0.78	0.08
No_PSO	Random Initialization	Improved Mutation Rule	Verify the Necessity of PSO Initialization	0.60	0.16
Hybrid_PSO	Hybrid Initialization (Random + PSO)	Improved Mutation Rule	Balance Initialization Diversity	0.62	0.14
Std_Mutation	PSO Initialization	Standard Polynomial Mutation	Validate the Effectiveness of the Improved Mutation	0.65	0.13
Fixed_Mutation	PSO Initialization	Fixed-parameter Mutation	Evaluate the Contribution of the Adaptive Mechanism	0.62	0.13
No_PSO_Std_Mut	Random Initialization	Standard Polynomial Mutation	Minimal Configuration (Without PSO or Improved Mutation)	0.51	0.20

As shown in Table 4, the improved NSGA-III algorithm achieves the highest average HV(0.78) and the lowest standard deviation(0.08), demonstrating superior performance in both solution quality and stability. When removing the BSA initialization strategy and using random initialization instead(No_PSO), the average HV drops significantly to 0.60, and the standard deviation rises to 0.16, indicating that chaotic initialization effectively improves the diversity and uniformity of the initial population, laying a solid foundation for global exploration and avoiding premature convergence caused by poor initial distribution. The hybrid initialization method(Hybrid_PSO) yields a slight improvement over pure random initialization but is still inferior to the full BSA initialization, confirming the necessity of the chaotic search mechanism for population initialization in this three-sided matching scenario.

When replacing the improved adaptive mutation with standard polynomial mutation(Std_Mutation) or fixed-parameter mutation(Fixed_Mutation), the average HV decreases to 0.65 and 0.62 respectively, accompanied by degraded stability. This result reveals that the designed adaptive mutation rule dynamically adjusts the perturbation amplitude according to the individual distribution in the solution space, strengthening global exploration in sparse regions and enhancing local exploitation in dense regions, thereby resolving the conflict between exploration and exploitation that commonly troubles traditional mutation operators. The baseline configuration without any improvements(No_PSO_Std_Mut) obtains the lowest HV(0.51) and the worst stability(Std HV = 0.20), which verifies the significant synergistic effect of the two improved strategies.

In summary, the BSA-based chaotic initialization and the adaptive mutation operator work complementarily to enhance the algorithm’s global searching ability, local optimization accuracy, and output stability simultaneously. Such improvements are particularly critical for solving the three-sided stable matching problem in organ transplantation, which is characterized by high dimensionality, multiple constraints, strong uncertainties, and heterogeneous preferences.

## 7. Conclusions

Aiming at the matching problem in the field of organ transplantation, this study constructs a three-sided stable matching model under a hybrid preference structure from the perspective of stable matching among three parties: organ recipients, medical institutions, and organ donors.

From a theoretical perspective, this study has constructed a three-sided stable matching model applicable to clinical scenarios. While balancing the interests of all sides, it has also improved the integrated handling mechanism for uncertainty, randomness, and ethical risks. Moreover, the improved NSGA-III algorithm designed in this paper integrates particle swarm initialization and adaptive mutation strategies, effectively enhancing the solution performance of multi-objective matching problems and providing a new solution approach for large-scale three-sided stable matching issues. Theoretical analysis and numerical experiments have shown that the proposed model and algorithm have good interpretability and operability, can significantly improve the matching efficiency and result stability, and further enrich the theory and method system in the field of medical resource allocation.

From a practical perspective, the three-sided stable matching framework proposed in this study can be directly applied to the clinical organ allocation and transplantation management system. It can simultaneously coordinate the decisions of donors, recipients, and medical institutions, thereby enhancing the fairness, stability, and efficiency of the allocation of scarce organ resources. By handling the uncertainty of medical indicators, the randomness of donor arrivals, and the deceptive behavior of recipients, the model is more in line with the actual clinical decision-making process. This framework is not only applicable to organ transplantation scenarios but can also be extended to similar allocation problems, such as cross-regional medical resource sharing. The improved NSGA-III algorithm can support the efficient solution of large-scale waiting lists. With simple parameter and objective adjustments, it can also be expanded to other medical scenarios such as ICU bed scheduling and surgical resource coordination, providing practical tools for clinical management and intelligent medical decision-making.

Although this study achieved relatively satisfactory results, there are still some limitations. Firstly, this paper uses a simulated data set instead of real clinical data, and there is still room for improvement in terms of data scale and diversity. Secondly, the model is quite sensitive to penalty coefficients, weight parameters, etc., and its robustness can still be enhanced. Finally, when dealing with ultra-large-scale real-time allocation tasks, there is still room for optimization in algorithmic computational efficiency. Future research will not only improve in the above aspects but also focus on information-based management of organ transplantation, design additional constraints for the potential game behaviors of matching participants, and further improve the efficiency and accuracy of organ transplantation management.

## Supporting information

S1 Data Minimum dataset. (XLSX)
